# Relationship of peak capillary blood lactate accumulation and body composition in determining the mechanical energy equivalent of lactate during sprint cycling

**DOI:** 10.1007/s00421-024-05529-9

**Published:** 2024-07-01

**Authors:** Benedikt Johannes Meixner, Valentin Nusser, Karsten Koehler, Mattice Sablain, Jan Boone, Billy Sperlich

**Affiliations:** 1 Integrative & Experimental Exercise Science & Training, Institute of Sport Science, Julius-Maximilians-Universität Würzburg, Judenbühlweg 11, 97082 Würzburg, Germany; 2 Department of Sport Science and Sport, Friedrich-Alexander-Universität Erlangen-Nürnberg, Gebbertstraße 123B, 91058 Erlangen, Germany; 3 Iq-Move Praxis Fraunberger, Gebbertstraße 123B, 91058 Erlangen, Germany; 4 Department of Health and Sport Science, TUM School of Medicine and Health, Technical University of Munich, Connollystr. 32, 80809 Munich, Germany; 5 Department of Movement and Sports Sciences, Ghent University, Watersportlaan 2, 9000 Ghent, Belgium

**Keywords:** Alactic, ATP, Energy equivalent, Gender difference, Performance

## Abstract

**Aim:**

A 15-s all-out sprint cycle test (i.e., νLa_max_-test) and the post-exercise change in capillary blood lactate concentration is an emerging diagnostic tool that is used to quantify the maximal glycolytic rate. The goal of this study was to determine the relation between 15 s-work, change in capillary blood lactate concentration (∆La) and body composition in a νLa_max_-test.

**Method:**

Fifty cyclists performed a 15 s all-out sprint test on a Cyclus2 ergometer twice after a previous familiarization trial. Capillary blood was sampled before and every minute (for 8 min) after the sprint to determine ∆La. Body composition was determined employing InBody720 eight-electrode impedance analysis.

**Result:**

Simple regression models of fat-free mass (FFM) and also the product of FFM and ∆La showed similar ability to predict 15 s-work (*R*^2^ = 0.79; 0.82). Multiple regression combining both predictors explains 93% of variance between individuals. No differences between males and females were found regarding 15 s-work relative to the product of fat-free mass and ∆La. Considering pairs of similar FFM, a change 1 mmol/l of ∆La is estimated to be equal to 12 J/kg in 15 s-work (*R*^2^ = 0.85).

**Discussion:**

Fifteen s-work is both closely related to FFM and also the product of ∆La and lactate-distribution space approximated by FFM. Differences in 15 s-work between males and females disappear when total lactate production is considered. Considering interindividual differences, the mechanical energy equivalent of blood lactate accumulation seems a robust parameter displaying a clear relationship between ∆La and 15 s-work relative to FFM.

## Introduction

The maximal rate of capillary blood lactate accumulation [νLa_max_] is a crucial element within Mader’s theoretical model designed to estimate the interplay between aerobic and anaerobic energy pathways at the cellular level (Mader 1984). This model also plays an important role in predicting athletic performance and elucidating metabolic variations (Mader 1984, 2003; Mader and Heck 1986; Wackerhage et al. 2022).

Within this model, the maximal glycolytic rate is typically evaluated using a 15 s all-out sprint test, accompanied by measuring capillary blood lactate levels in a passive resting state both before and after the sprint. (Heck et al. 2003; Adam et al. 2015; Nitzsche, et al. 2018; Quittmann et al. 2021a, b; Quittmann et al. 2021a, b).

Early findings by Margaria and coauthors (Margaria et al. 1963, 1964) provide the basis for an assumption of the energetic oxygen equivalent for lactate concentration during treadmill sprinting in connection with post-exercise peak capillary blood lactate concentration (di Prampero and Ferretti 1999). The authors directly related changes in capillary blood with work output and note a linear relationship.

To date, existing research on post-exercise peak capillary blood lactate concentration has primarily focused on assessing its reliability (Adam et al. 2015; Quittmann et al. 2021a, b; Held, et al. 2023) or the effectiveness of Mader’s model as a predictive measure for endurance performance (Hauser et al. 2014; Ji et al. 2021; Quittmann et al. 2022). Although there is a recognition of νLa_max_ and its relationship to power outputs, the potential for a direct (i.e., mechanical) energy equivalent of capillary blood lactate production in connection with sprint cycling has not been investigated so far. Investigating this relationship, specifically the capillary blood lactate levels post-sprint cycling relative to the work output, could facilitate the establishment of a linear work-to-lactate energy equivalence and thereby improve sprint-cycling performance prediction.

In addition to the metabolic contributions of glycolysis in sprint cycling, research has also highlighted the impact of body composition, particularly fat-free mass (FFM), on sprint-cycling performance (Vardar et al. 2007). Females in general display less FFM and a higher percentage of body fat which at least in part may explain the differences in peak power output and total work during sprint cycling between men and women. The majority of FFM consists of skeletal muscle, which is the primary tissue for lactate production during heavy exercise (Brooks 2007). As muscle mass increases, the potential for total lactate production and the lactate-distribution space as fraction of body water—both proportionally related to FFM—also increase (Wang et al. 1999). Since one feature of Mader’s model is to estimate cellular metabolic processes based on capillary blood lactate measurement, the model assumes a fraction of total body water as variable for the dilution space of blood lactate outside of the muscle cell (Mader and Heck 1986). From this perspective it is of interest how body composition, especially FFM, affects νLa_max_ between male and female sprint cyclists.

Based on the aforementioned information the aim of this study was threefold:

(i) to identify the relationship of FFM, maximal glycolytic rate and 15 s-work, (ii) to compare the relation of 15 s-work and capillary blood lactate accumulation for both males and females, (iii) to assess the mechanical energy equivalent of capillary blood-lactate accumulation, i.e., the amount of energy that can be derived from lactate production during 15-s all-out cycle sprinting.

We hypothesized that: (i) FFM and capillary blood-lactate accumulation are positively correlated with 15 s-work, (ii) while males and females show differences in absolute and relative 15 s-work, no disparities are observed when considering blood lactate accumulation as a marker of glycolysis, and (iii) work output exhibits a near-linear relationship with blood-lactate accumulation, aligning closely with findings by Margaria et al. during treadmill sprinting (di Prampero and Ferretti 1999).

## Methods

### Participants

A cohort of *n* = 50 (*n* = 30 male, *n* = 20 female) experienced cyclists with more than three years of regular cycling exercise (> 2 sessions per week) were recruited for this study. All participants were experienced in road cycling with clipless pedals and cycled regularly as exercise. Prior to the study, the participants were informed of the protocol and gave their written informed consent to participate. All procedures were approved by the ethical committee of Exercise Science & Training of the Faculty of Human Sciences of the University of Würzburg (EV2024/1-1004) and conducted in accordance with the Declaration of Helsinki (Harriss and Atkinson 2009; World Medical Association 2013). Characteristics of participants are given in Table 1.

**Table 1:** Mean ± SD of age, body stature, selected anthropometric data and peak oxygen uptake of participants obtained during the first visit

Variable	Female (*n* = 20)	Male (*n* = 30)	All (*n* = 50)
Age [years]	26.5 ± 4.9	34.4 ± 7.8*	31.2 ± 7.8
Height [cm]	169.8 ± 6.6	182.6 ± 7.0*	177.5 ± 9.3
Body mass [kg]	61.4 ± 6.6	78.6 ± 10.3*	71.7 ± 12.4
Maximum oxygen uptake [ml/kg/min]	52.4 ± 5.9	57.4 ± 8.0*	55.4 ± 7.6

*Indicates unpaired *t*-test differences between male and female

### Experimental design

**Fig. 1:**
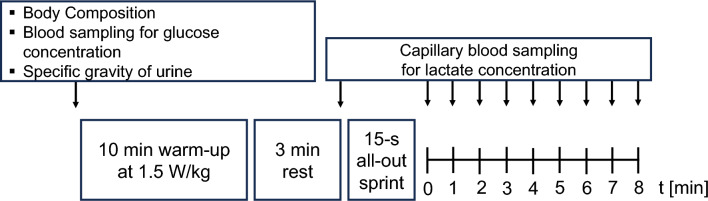
Illustration of the timeline of all study procedures

Three experimental visits to the laboratory were required which were at least 48 h apart and completed within a period of two weeks. The first visit was to familiarize participants with the all-out sprint and determine their ${\ddot {\text{V}}}$O_2peak_ in a ramp test. Figure 1 illustrates the timeline and all testing procedure for each visit.

All participants were instructed to keep a nutrition diary and to repeat their usual diet for each visit within the 24 h before each experimental visit (Jeacocke and Burke 2010). In addition, all were instructed to stay adequately hydrated, to eat a carbohydrate-rich meal (i.e., a banana and a jam sandwich) no less than 3 h before each visit and to refrain from caffeine consumption on the day. Each participant received 35 g of a carbohydrate mixture (IsoFast, DextroEnergy, Krefeld, Germany) dissolved in 500 ml of water to drink ad libitum during warm-up and recovery periods. All participants provided a urine sample in a cup first when visiting the laboratory. Hydration status was then promptly tested via urine specific gravity analysis employing a dipstick (One step 10, DFI Co., Gyeongsangnam-do, South Korea).

During the first visit (T1), body composition (i.e., FFM) of all participants was measured employing eight-electrode impedance analysis (InBody 720, Biospace, Des Moines, Iowa, USA). During the second (T2) and third (T3) visit, only a warm-up and 15-s all-out cycle sprint test were performed.

All 15-s all-out cycle sprints were conducted on their own personal road bike installed on a Cyclus2 ergometer (RBM, Leipzig, Germany). The Cyclus2 is an electromagnetically braked ergometer and measures power with an accuracy error of 2% according to the manufacturer. All cyclists used their own shoes and pedals for all tests. For all three visits, all cyclists warmed up for 10 min cycling at 1.5 W/kg body mass and resting for 3 min (Quittmann et al. 2021a, b).

The all-out cycle sprint was performed in a seated position utilizing the large chainring (if applicable) of the participant’s bike and the 15-tooth cog of the ergometer. Recording of the test started with cadence of > 30 RPM. The ergometer software was set to isokinetic mode and 130 RPM (Adam et al. 2015; Nitzsche, et al. 2018; Quittmann et al. 2020; Quittmann et al. 2021a, b).

Capillary blood samples of the left earlobe were sampled twice during the resting period, directly after the warm-up while resting passively and once directly after the sprint as well as every minute for 8 minutes after the 15 s cycle sprint. Lactate concentration was measured amperometric-enzymatically employing Biosen C-Line (EKF Diagnostics, Barleben, Germany). Peak lactate was taken as the highest measured concentration during the passive rest period. ∆La was calculated as the difference between the average of resting values (La_pre_) and the peak values attained in the post-exercise resting period (La_peakpost_).

Approximately 30 min after the sprint test, V̇̇O_2peak_ was determined in a ramp test protocol. Participants were fitted with a Hans Rudolph V2 mask (Hans Rudolph, Inc, Shawnee, KS, USA) and expired gasses and breathing volume were analyzed with Cosmed Quark CPET (Cosmed Srl, Rome, Italy). The gas and volume analyzers were calibrated before every test with precision gas (16% O_2_; 5% CO_2_) and a volume pump in accordance with the manufacturer's instructions (Airgas Therapeutics, Plumsteadville, PA, USA). Participants cycled at 100 W for 2 min with freely chosen cadence. Thereafter the load increased by 25 W every 30 s (Adam et al. 2015). The test ended when volitional exhaustion was reached or cadence dropped by more than 10 RPM. V̇O_2peak_ was calculated as the highest value averaged over 30 s.

### Mechanical energy equivalent of lactate

As result of the previously shown high reliability of the testing procedure for 15 s-work (ICC = 0.99) and ∆La (ICC = 0.91), 15 s-work and ∆La was averaged from T2 and T3 to account for day-to-day variability (Zinner et al. 2023; Meixner et al. 2024). T1 was considered as familiarization because of decreased reliability compared to T2 and T3.

The calculation of glycolytic energy contribution was based on previous work (Margaria et al. 1963, 1964). An oxygen equivalent of 3 ml O_2_ per kg bodyweight was assumed per 1 mmol/l of accumulated lactate in capillary blood blood. This oxygen equivalent was transformed to work assuming an energy equivalent of 21.1 kJ/l O_2_ and gross efficiency of 20% (Scott 2005).

As total body water is a constant in humans of 73% of FFM (Wang et al. 1999) and Mader and Heck consider 69% of total body water to be available as lactate-distribution space (Mader and Heck 1986), lactate-distribution space is ≈ 0.5*FFM. In the present study, an equivalent measure to the method of Margaria was calculated multiplying ΔLa with lactate-distribution space as assumed total lactate production. An oxygen equivalent of 7 ml O_2_ per kg FFM per change of mol of capillary blood lactate accumulated was determined to be comparable to the method of Margaria via linear regression. The resulting value is referred to as glycolytic energy contribution.

Considering the apparent influence of FFM and total lactate production, we conducted a post-hoc analysis to identify all possible pairs of participants whose FFM differed by no more than 1.5%. For these pairs (*n* = 51), the impact of FFM on differences in 15 s-work output was deemed negligible. Subsequently, we calculated the mechanical energy equivalent of blood-lactate accumulation by relating the difference in relative 15 s-work per kilogram of FFM between pairs to the difference in ΔLa for each pair.

### Statistical analyses

Raw data was processed using Microsoft Excel. Statistical analyses (mean, standard deviations, and 95% confidence intervals) were computed with GraphPad Prism (v10.2, Boston, MA, USA). Data normality for body mass, FFM, 15 s-work (absolute and relative to FFM) and ΔLa was assessed using the Kolmogorov–Smirnov test, Shapiro–Wilk test and visual inspection, without requiring further transformation. Level of significance (*α*) was set to 0.05 for all statistical analysis. Relations between 15 s-work, ΔLa, FFM and associated measures were analyzed employing simple and multiple linear regression models. Differences between males and females were analyzed by independent sample student *t* tests.

## Results

**Fig. 2:**
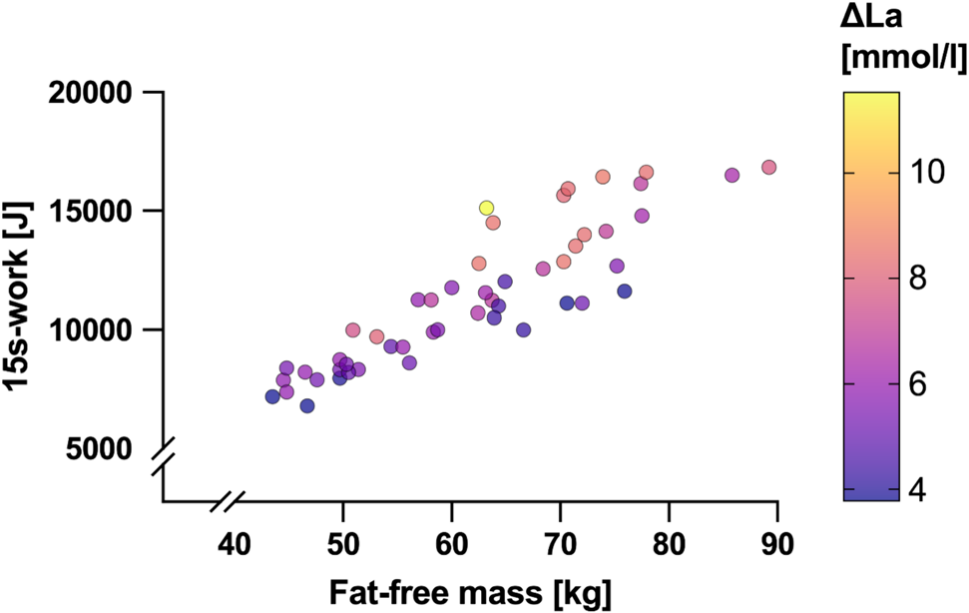
Illustration of relation of total work, Fat-free mass and ΔLa

The results are shown as mean ± SD in Table 2 and individually graphed in Fig. 2.

**Table 2:** Mean ± SD of 15 s-work, ΔLa, 15 s-work relative to fat-free mass, total lactate accumulation of T2 and T3 averaged

Variable	Female (*n* = 20)	Male (*n* = 30)	All (*n* = 50)
15 s-work [J]	8654 ± 1258	13,111 ± 2246	11,328 ± 2909
ΔLa [mmol/l]	5.60 ± 1.09	6.70 ± 1.81	6.26 ± 1.64
15 s-work/FFM [J/kg]	170 ± 12	189 ± 24	182 ± 22
Total lactate accumulation [mmol]	143 ± 38	232 ± 70	197 ± 73
Fat-free mass [kg]	50.8 ± 5.8	69.2 ± 7.9	61.9 ± 11.5
Body fat [%]	17.1 ± 4.2	11.7 ± 3.8	13.9 ± 4.8
Resting La	0.84 ± 0.19	0.86 ± 0.21	0.85 ± 0.20
Peak La	6.43 ± 1.10	7.56 ± 1.87	7.11 ± 1.69

**Fig. 3:**
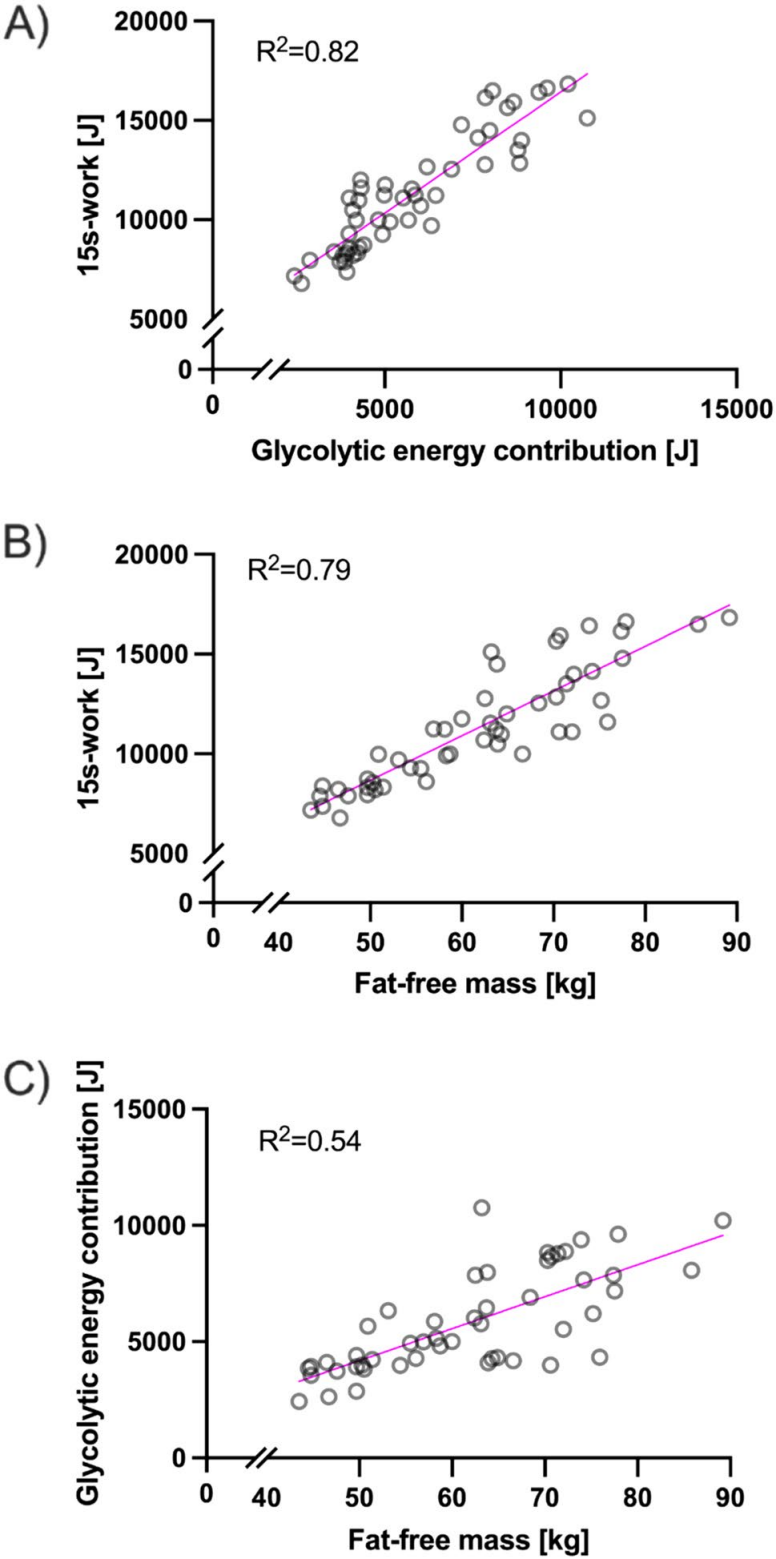
Relation of **A** 15 s-work to glycolytic energy contribution **B** 15 s-work to fat-free mass **C** Collinearity of fat-free mass and glycolytic energy contribution

The glycolytic energy contribution calculated considering a fraction of FFM mass instead of total bodyweight is correlated to 15 s-work during the sprint test (*R*^2^ = 0.82, *p* < 0.01, Fig. 3A). FFM also (*R*^2^ = 0.79, *p* < 0.01, Fig. 3B) emerged as predictor for 15 s-work. Collinearity between FFM and glycolytic energy contribution is moderate (*R*^2^ = 0.54, Fig. 3C).

A multiple linear regression model incorporating both FFM and glycolytic energy contribution can be used to explain 15 s-work (*R*^2^ = 0.93, *p* < 0.01). The fitted equation was calculated by the regression model as follows:

$$15- \text{s work }=-551+123*FFM+0.74*glycolytic \,energy \,contribution$$

**Fig. 4:**
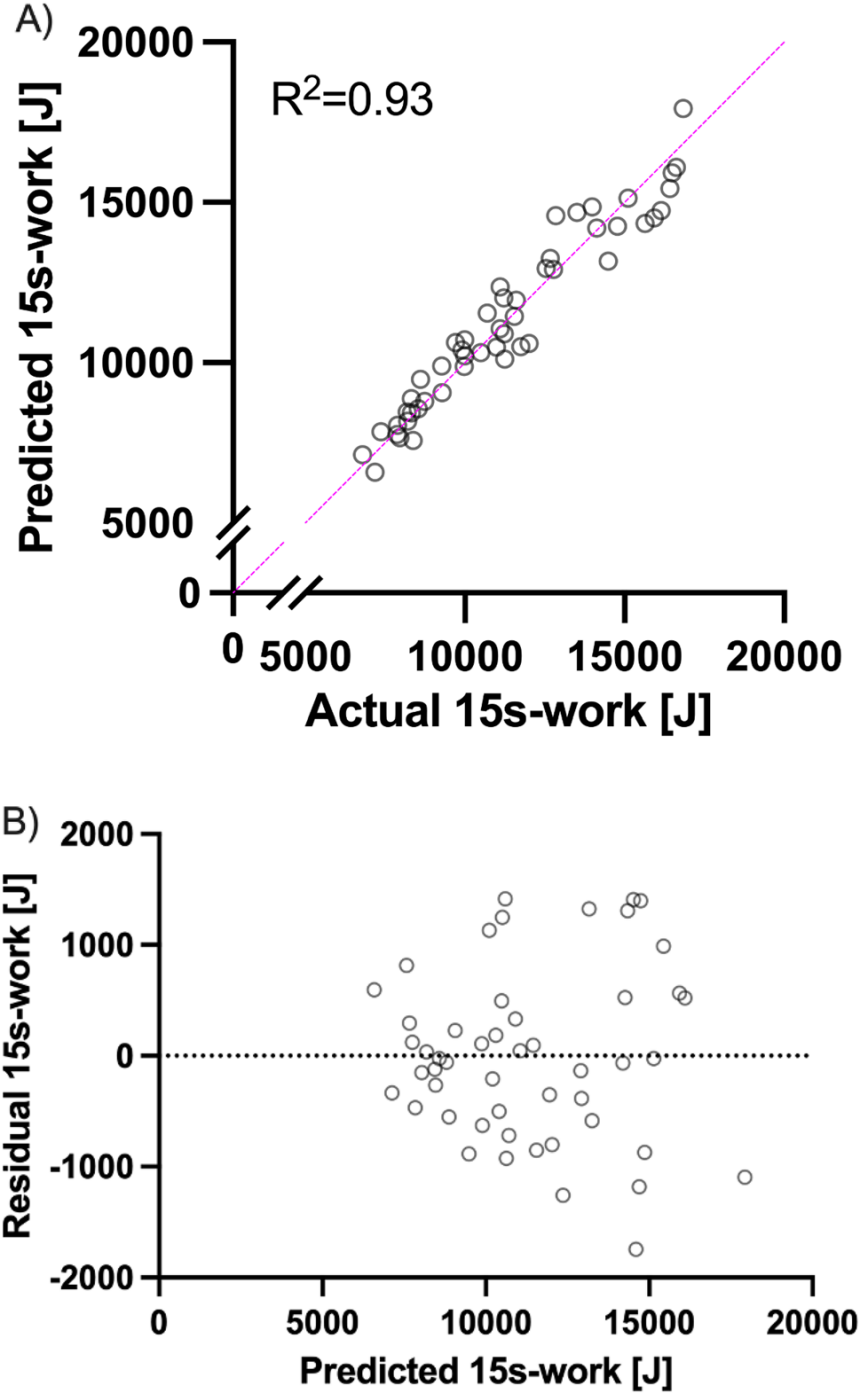
**A** Comparison of actual 15 s-work and predicted 15 s-work and **B** Residual plot for the multiple linear regression model

Both covariates attained statistical significance (p < 0.0001). The correlation of actual 15 s-work and 15 s-work predicted by the regression model as well as the residual plot can be seen in Fig. 4A and B.

**Fig. 5:**
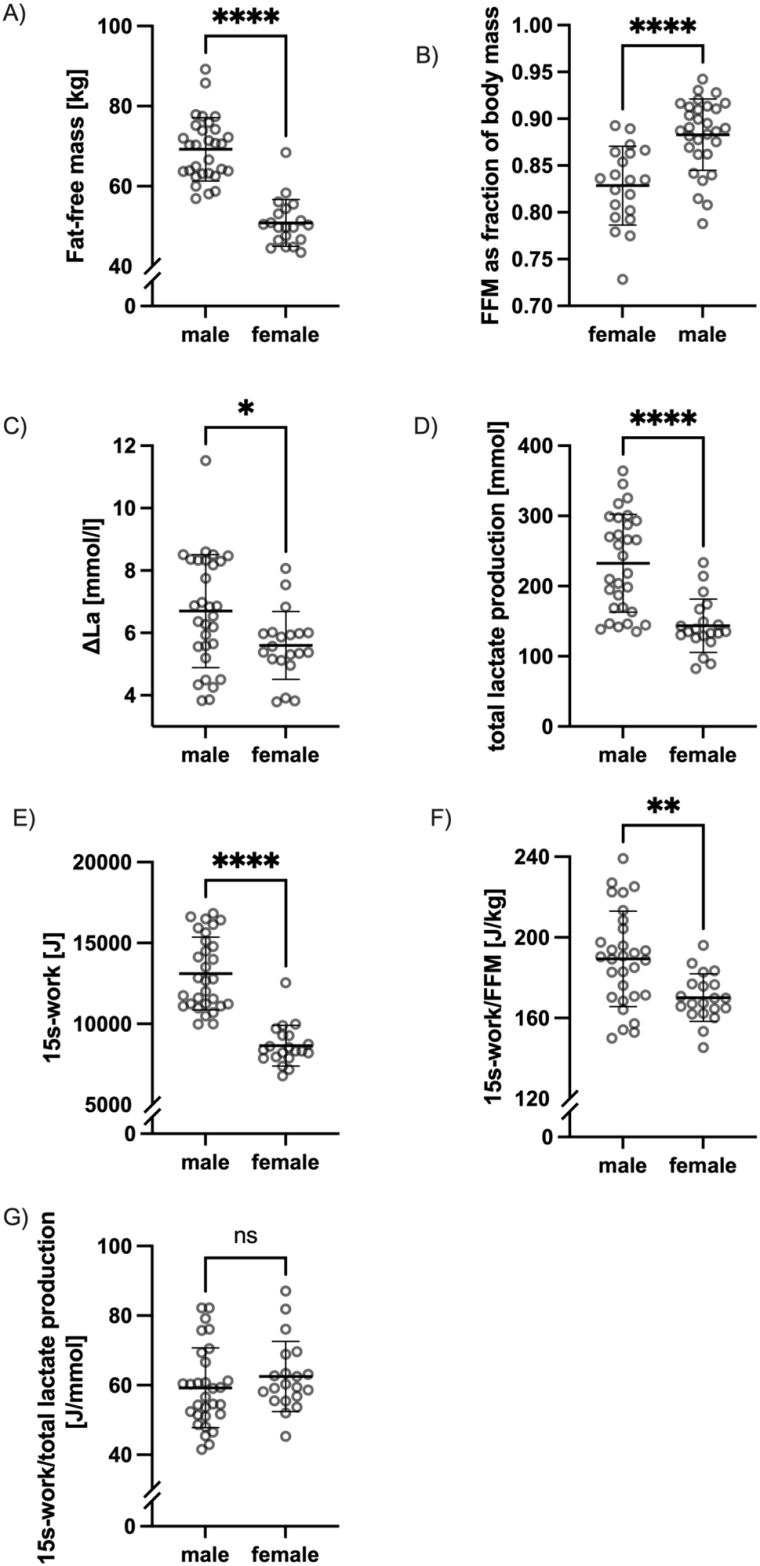
Differences between mean ± SD between male and female groups for **A** fat-free mass **B** fat-free mass as fraction of body mass **C** ΔLa **D** 15 s-work **E** total lactate production **F** 15 s-work relative to FFM **G** 15 s-work relative to total lactate production

Unpaired student *t* tests revealed significant differences between groups for work, FFM, work normalized to FFM, ΔLa and total lactate accumulation. No statistical difference was found for the relation of work normalized to lactate production (i.e., ΔLa * FFM). Graphical representations for relevant variables are shown in Fig. 5A–F.

**Fig. 6:**
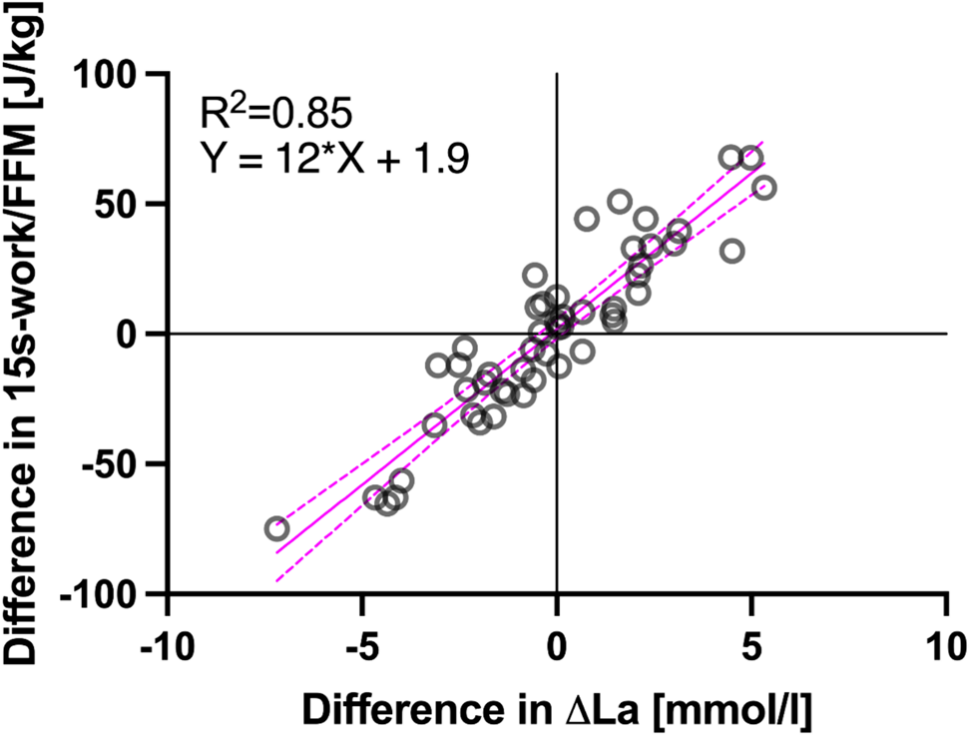
Differences between ΔLa and 15 s-work relative to FFM in each pair of less than 1.5% difference in FFM

Differences in 15 s-work and ΔLa in pairs with no more than 1.5% difference in FFM (*n* = 51) show a significant relation between Work and ΔLa (*R*^2^ = 0.85, *p* < 0.01). Regression analysis equates a difference in 1 mmol of blood-lactate accumulation to a difference of 12 J/kg FFM of work during the sprint. This is depicted graphically in Fig. 6.

## Discussion

The main findings of the present study are as follows:

i) 15 s-work in an all-out cycle sprint is largely explained by i) FFM and ii) glycolytic energy contribution;
ii) No differences exist between males and females for the relation between 15 s-work and total lactate accumulated;
iii) The mechanical energy equivalent of 1 mmol/l of blood-lactate accumulation is equated to 12 J/kg FFM of 15 s-work.

### Components of 15 s-work

Previous studies have employed the difference between resting and peak post-exercise lactate levels, i.e., ΔLa for predicting work-output during sprinting (Zwingmann 2020; Held et al. 2023; Mavroudi et al. 2023; Langley et al. 2024). However, based on our data a simple linear regression model only employing ΔLa is a poor predictor for 15 s-work. One reason for the divergent results is attributable to differences in the dilution space of lactate. Since it is known that only parts of FFM act as the dilution space of blood lactate outside of the muscle cell (Mader and Heck 1986) we added FFM to the regression analysis to understand the relationship of blood-lactate accumulation and 15 s-work in more detail. In line with the single compartment model of Mader and Heck (Mader and Heck 1986), we further assessed the prediction of 15 s-work by glycolytic energy contribution. This approach mirrors the calculation method of Margaria et al. but enhances it by exclusively considering FFM rather than total BM. (Margaria et al. 1964; di Prampero and Ferretti 1999). In this case the regression provided an *R*^2^ = 0.82 (*p* < 0.0001) for this measure to work output (Fig. 3A).

Interestingly, a linear regression using only FFM as a predictor of 15 s-work showed comparable explanation of variance (*R*^2^ = 0.79, *p* < 0.0001), casting doubt on the effectiveness of adding ΔLa to calculations to predict 15 s-work (Fig. 3B). The collinearity between FFM and lactate accumulation over FFM is only moderate (*R*^2^ = 0.54), suggesting they may be considered independent predictors. Therefore, in a next step we employed a multiple linear regression model aiming to assess the prediction of work output employing FFM and glycolytic energy contribution because of two reasons:

- (i) whole body muscle mass and lower limb mass are predictors of sprint performance in cycling (Martin et al. 2007; Perez-Gomez et al. 2008);
- (ii) other studies (Yang et al. 2023) have estimated that phosphagen contributes approximately 61.8 ± 7.6% to work in a 15 s sprint. Phosphocreatine is primarily found in skeletal muscle (Kreider and Stout 2021). Increased muscle mass is therefore expected to increase creatine content of the whole body and increase the amount of work by phosphagen contribution. In addition, the cross-sectional area of working muscles is linked to maximal force production (Douglas et al. 2021). Integrating these insights, increased muscle mass not only serves as a reservoir for phosphocreatine but also potentially enhances the amount of work provided via this energy pathway.

Our data show that FFM combined with the glycolytic energy contribution (i.e., the product of ΔLa and a fraction of FFM as a proxy for total lactate production), can predict work output during a 15 s cycling sprint with *R*^2^ = 0.93 (*p* < 0.0001, Fig. 4A). Consequently, FFM along with glycolytic energy contribution proves to be a robust predictor of 15 s-work in both male and female cyclists.

The β value for the glycolytic energy contribution in the regression model is less than 1 (0.74, with a 95% CI 0.58 to 0.89), suggesting that the contribution of glycolysis may be less significant than initially calculated. Alternatively, this finding could indicate that sprint performance is influenced not just by energy dynamics but also by mechanical factors as it has been proposed earlier (Bundle and Weyand 2012). Lactate production is ultimately the consequence of a short-term challenge to ATP because of the body’s metabolic response to high-intensity conditions where energy demands exceed the immediate availability of oxygen (Brooks 2018). It remains unclear whether sprint performance is limited by the glycolytic contribution or ATP demand is limited by other factors such as neuromuscular activation. Bundle and Weyand provide arguments for metabolic energy release being demand-driven and not supply-limited (Bundle and Weyand 2012). According to their theory, sprint and endurance exercise differ greatly: endurance performance is limited mainly by available metabolic energy while sprint performance is determined by the intensity of mechanical activity and properties achieved by the neuromuscular system. This perspective adds to the controversy about the validity of νLa_max_ as a metabolic marker. In this case, blood-lactate accumulation seems a consequence of a high ATP demand, which itself is limited by the cyclist’s ability to produce muscular force during sprinting.

### Sex differences

To our knowledge, the present study is the first to include a large number of female cyclists in 15-s sprint cycling and the evaluation of the maximal accumulation rate of capillary blood lactate. As in other studies (Mayhew and Salm 1990; Perez-Gomez et al. 2008; Ferguson et al. 2023), the 15 s-work of males was found to be higher than the corresponding values of females. This also remains the case when normalizing the power data to FFM. Women also exhibit lower changes in blood-lactate values, both in concentration and when considering the size of distribution space, as shown before in studies investigating 30-s all-out cycling (Esbjörnsson-Liljedahl et al. 2002).

Muscle-fiber typology of the different sexes may be one factor explaining the differences in capillary blood-lactate concentration and power output. In general, women possess lower proportions of type II muscle fibers (Esbjörnsson-Liljedahl et al. 2002) which are associated with increased glycolysis and glycogenolysis.

When considering power output per total lactate production, we found no difference between male and female cyclists (Fig. 6G). Although the ability to produce lactate as well as lean body mass seems one obvious sex difference in sprint cycling, the present results indicate that the amount of work output per mmol of lactate is the same for both male and female cyclists.

### Mechanical energy equivalent of lactate accumulation

We found no significant statistical differences between males and females in the amount of work relative to lactate production. Therefore, the applied calculation for the mechanical energy equivalent of blood-lactate accumulation did not distinguish between males and females.

Based on the present data the mechanical energy equivalent of 1 mmol/l blood-lactate accumulation is 12 J/kg of FFM for the total duration of the 15 s all-out cycle sprint. This value is evident by the slope of 12 (95% CI 10.5 to 13.4) of our regression model. To provide a context to these findings, the assumed metabolic energy equivalent of Margaria et al. was 3 ml O_2_/kg BM per mmol/l of lactate accumulation (Margaria et al. 1964). This value was originally determined for treadmill running at different inclines (Margaria et al. 1964). Assuming 21.1 kJ/l O_2_ and efficiency of 20%, this amounts to ≈ 12.7 J/kg of BW (Scott 2005). In contrast to the findings of Margaria et al. (Margaria et al. 1964), our mechanical energetic equivalent was obtained using the difference between pairs of roughly equal FFM instead of in the same individual and different work outputs with no transformation. For cycling, an oxygen equivalent of 2.8 ml O_2_/kg BW was determined previously, which equals 11.8 J/kg of BW under the same assumptions (di Prampero and Ferretti 1999; Ferretti 2015, 2023). Including several assumptions and approximations for distribution of lactate, di Prampero and Pendergast calculated 85.7 kJ/mol of lactate in terms of metabolic energy (Di Prampero, Pendergast et al. 1978). This results in 17.1 J/mmol of lactate in terms of actual power output when assuming 20% efficiency.

Considering interindividual factors, the linear relationship of the differences in ΔLa and work output between pairs of similar FFM seems remarkably robust (*R*^2^ = 0.85). Individuals may exhibit different cycling efficiencies, altered distribution of FFM among legs, arms and trunk as well as lactate kinetics related to elimination, distribution, and transport of lactate. In light of the previous discussion (di Prampero and Ferretti 1999; Gastin 2001; Ferretti 2015, 2023) on the concept of equilibrium in lactate distribution, we interpret our results as evidence that capillary blood lactate may provide a quantifiable estimate of energy release (di Prampero and Ferretti 1999; Ferretti 2015). The energy equivalent of blood lactate should be treated with caution due to individual variations, which can significantly affect its accuracy and reliability (Hill and Mihalek 2024).

### Strengths and limitations

We consider the sample size (*n* = 50) and the inclusion of a large group of females (*n* = 20) as considerable strengths of this study. One further aspect is the use of the individuals own bike, which we deem a necessity for this type of testing.

The findings of this study cannot offer new insights into the conundrum of high lactate production as either requisite or consequence of high work outputs in a sprint (Bundle and Weyand 2012; Wackerhage et al. 2022). Further studies may investigate this problem by employing muscle biopsies and EMG measurements.

The mechanical energy equivalent of blood-lactate accumulation was determined by identifying pairs of similar FFM. Further confirmation of this energetic equivalent may be obtained by determining intra-individual differences in blood-lactate accumulation and power output, possibly by altering the pre-set power outputs and/or revolutions per minute. Individual discrepancies in efficiency, blood-lactate kinetics and distribution of FFM would be rendered irrelevant under these conditions. Also, the role of body composition may be different when employing another method for body composition.

Glycolysis is only one energetic pathway that contributes to work output in a 15 s all-out sprint. The main energetic contributor to work output is the anaerobic alactic pathway of ATP and CrP (Yang et al. 2023). Changes in the energetic contribution of this pathway cannot be measured by our employed method but may influence work output significantly. While often thought to be negligible, oxidative phosphorylation plays a role in energetic contribution in a 15 s sprint and differs between subjects (Yang et al. 2023). This was not measured and therefore also not considered in our study.

## Conclusion

Along with body composition, blood-lactate accumulation is (i) a strong predictor for work output during a 15 s all-out sprint (ii) has a mechanical energy equivalent of 12 J/kg per mmol and (iii) does not differ between males and females.

These findings have potential applications for interpretation of sprint testing, refining simulation models of metabolism and performance-enhancing interventions for cyclists.

## Data Availability

The data that support the findings of this study are available on reasonable request from the corresponding author. The data are not publicly available due to privacy or ethical restrictions.

## Abbreviations

∆La: Difference in lactate concentration from La_pre_ to La_peakpost_
ATP: Adenosine-triphosphate
BM: Body mass
CrP: Phosphocreatine
CI: Confidence interval
FFM: Fat-free mass
ICC: Intraclass correlation coefficient
La_peakpost_: Peak lactate concentration during the recovery period
La_pre_: Mean value of two capillary blood lactate samples before sprint test
PFK: Phosphofructokinase
RPM: Revolutions per minute
T1: First experimental visit
T2: Second experimental visit
T3: Third experimental visit
νLa_max_: Maximal rate of change in capillary blood lactate concentration
${\ddot {\text{V}}}$O_2__peak_: Peak oxygen uptake
